# Disruption of Murine *mp29/Syf2/Ntc31* Gene Results in Embryonic Lethality with Aberrant Checkpoint Response

**DOI:** 10.1371/journal.pone.0033538

**Published:** 2012-03-20

**Authors:** Chia-Hsin Chen, Po-Chen Chu, Liekyeow Lee, Huang-Wei Lien, Tse-Ling Lin, Chi-Chen Fan, Peter Chi, Chang-Jen Huang, Mau-Sun Chang

**Affiliations:** 1 Institute of Biochemical Sciences, College of Life Science, National Taiwan University, Taipei, Taiwan; 2 Institute of Fisheries Science, College of Life Science, National Taiwan University, Taipei, Taiwan; 3 Institute of Biological Chemistry, Academia Sinica, Taipei, Taiwan; 4 Department of Physiology, Mackay Memorial Hospital, Taipei, Taiwan; Montana State University, United States of America

## Abstract

Human p29 is a putative component of spliceosomes, but its role in pre-mRNA is elusive. By siRNA knockdown and stable overexpression, we demonstrated that human p29 is involved in DNA damage response and Fanconi anemia pathway in cultured cells. In this study, we generated *p29* knockout mice (*mp29^GT/GT^*) using the *mp29* gene trap embryonic stem cells to study the role of *mp29* in DNA damage response *in vivo*. Interruption of *mp29* at both alleles resulted in embryonic lethality. Embryonic abnormality occurred as early as E6.5 in *mp29^GT/GT^* mice accompanied with decreased mRNA levels of α-tubulin and Chk1. The reduction of α-tubulin and Chk1 mRNAs is likely due to an impaired post-transcriptional event. An aberrant G2/M checkpoint was found in *mp29* gene trap embryos when exposed to aphidicolin and UV light. This embryonic lethality was rescued by crossing with *mp29* transgenic mice. Additionally, the knockdown of zfp29 in zebrafish resulted in embryonic death at 72 hours of development postfertilization (hpf). A lower level of acetylated α-tubulin was also observed in zfp29 morphants. Together, these results illustrate an indispensable role of *mp29* in DNA checkpoint response during embryonic development.

## Introduction

Gene expression is regulated by a series of events taking place at both the transcriptional and post-transcriptional levels through mechanisms of pre-mRNA splicing, mRNA stability and mRNA transport. In eukaryotes, introns must be removed from precursor mRNA (pre-mRNA) by the spliceosome. U1, U2, U4, U5, and U6 small nuclear ribonucleoproteins (snRNPs) are the main elements of the spliceosome responsible for removing the majority of pre-mRNA introns. Moreover, each snRNP consists of one or two small nuclear RNAs (snRNAs) and many associated proteins, namely pre-mRNA processing proteins (Prp). Mutantions in *Prp* have been shown to be defective in removal of pre-mRNA introns [1].

*Syf2/Ntc31*, a putative homolog of human *p29* in *Saccharomyces cerevisiae*, was independently identified as a synthetic lethal mutant accompanied with the absence of *PRP17/CDC40* gene and a component of the Prp19p-associated complex [2], [3]. Although mutation in *SYF2/NTC31* has no apparently aberrant cell cycle phenotype, lower levels of U5 and U6 snRNPs were pulled down with Syf1p/Syf3p/Isy1p in *SYF2* deletion mutant [4]. Deletion of two non-essential splicing factors *ISYL1 and SYF2* shows a reduced expression of α-tubulin at the restrictive temperature, which results in the G2/M cell cycle arrest [5]. This indicates that Syf2 and Isy1 are synergenically responsible for *TUB1* and *TUB3* pre-mRNA splicing in yeast. Clf1 is a conserved spliceosome assembly factor with tetratricopeptide repeats (TPR). Clf1 has been shown interact with Syf2 from the results of yeast two-hybrid analyses and protein-binding assays [6]. Mass spectrometry analyses of human spliceosomes reveal that human p29/syf2/Ntc31p associates with Prp19 and appears in the complex B/C of spliceosomes [1], [5], but the precise role of p29 in pre-mRNA splicing is unclear.

In addition to its putative involvement in pre-mRNA splicing, we have found that human p29 plays an important role in the regulation of DNA damage response (DDR) [7]. It is well known that cells will exploit DDR to maintain their genome integrity upon genotoxic stress. Formation of double strand breaks activates poly(ADP-ribose) polymerase 1 (PARP1), which recruits the Mre11-Rad50-Nbs1 (MRN)/ATM complex to the damaged sites. Subsequently, activation of ATM kinase leads to phosphorylation of downstream targets, such as Chk2 and p53. By contrast, DNA lesions induced by replication stress or UV irradiation result in the accumulation of RPA-coated ssDNA and loading of ATR-ATRIP and Rad9-Rad1-Hus1 (9-1-1) complex. Stimulation of ATR kinase by the 9-1-1 associated protein TopBP1 activates ATR signaling cascade, including downstream Chk1 phosphorylation [8]. We have reported that depletion of human p29 downregulated Chk1 phosphorylation upon UV irradiation and resulted in premature chromatin condensation which initiated apoptosis [7]. Analyses of U2OS cells with constitutive p29 expression revealed increased phosphorylation levels of Chk1 and Chk2. Moreover, the monoubiquitination of FANCD2 and FANCI complex was restored in Fanconi anemia complementation group G (FA-G) cells stably expressing p29 [9]. We further established *mp29* transgenic mice under the control of mouse *PGK1* promoter. The elevated expression of mp29 protein in *mp29^Tg/+^* heterozygous mice was confirmed in the tissue extracts prepared from the brain, kidney, spleen, liver and testis by immunoblotting. Additionally, lower tumor incidence was found in *mp29* transgenic mice after UV irradiation [9]. However, an *mp29* deficient mouse model has not been established yet.

To investigate the function of *mp29 in vivo*, we generated *mp29* deficient mice using a gene trap ES cell line. Molecular examinations of *mp29^GT/GT^* mutant embryos, a complementation test with *mp29* transgenic mice, and morpholino (MO) knockdown of *zfp29* in zebrafish indicate that mp29/syf2/Ntc31 plays a crucial role for the survival of embryos.

## Results

### Generation of *mp29* knockout mice

**Figure 1:**
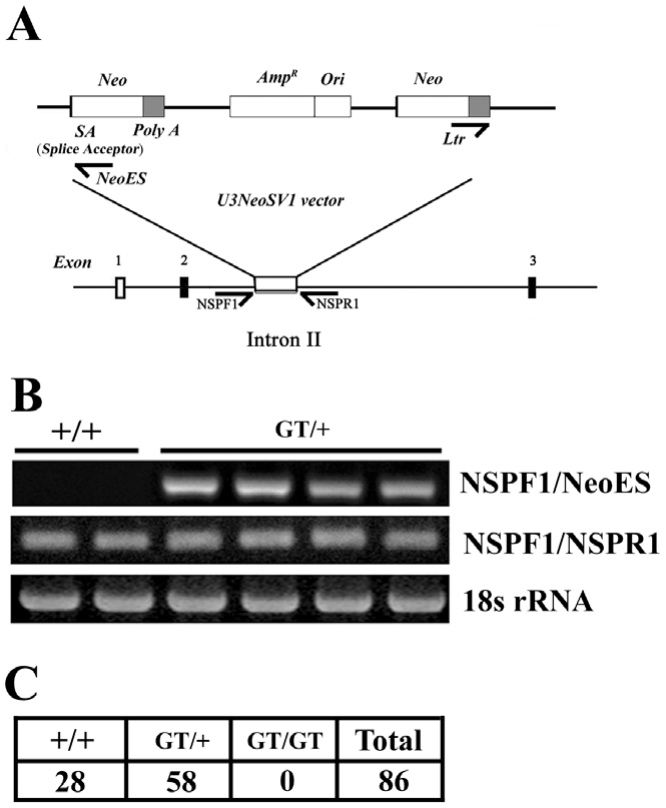
Interruption of mouse *mp29* gene by a gene trap vector. (A) Schematic of the targeting vector U3NeoSV1 within exon 2 and 3 of *mp29* gene. (B) Genotyping of mouse tail DNAs by PCR. NSPF1/NeoES primers were used to examine the presence of U3NeoSV1 vector in *mp29* gene with a product of 250 bp. NSPF1/R1 primers were used to determine if both of alleles were inserted with U3NeoSV1 vectors with a product of 300 bp. 18s RNA was used as a control. +/+: Wild type mice; GT/+: Heterozygous mice with U3NeoSV1 inserted in one allele. (C) Of 86 offspring mice, 28 pups were identified as wild type (+/+) and 58 pups were heterozygous (GT/+).

Mouse mp29 shares approximate 13% of amino acid identity with yeast Syf2 (Figure S1 for the first supporting information figure). The wild-type murine *mp29* gene locates on chromosome 4 and contains 7 exons [10]. The location of a gene trap vector was inserted between exon 2 and 3 on *mp29* gene (Fig. 1A). Chimeras capable of germ-line transmission were backcrossed to strain C57BL/6 mice to generate *mp29* gene-trap heterozygous mice (*mp29^GT/+^*), which were in the 129Sv2/C57BL6 mixed background. Adult heterozygous mice were healthy and fertile. Male and female *mp29^GT/+^* mice were intercrossed to produce *mp29* gene-trap homozygous mice (*mp29^GT/GT^*). PCR analyses were used to determine the genotypes of the offsprings at age of 4 weeks (Fig. 1B). Out of 86 progeny analyzed, *mp29^+/+^* and *mp29^GT/+^* mice were approximately in a ratio of 1∶2 and no significant phenotypic differences were found among these mice. Nevertheless, we did not detect any mice inherited with two interrupted alleles (Fig. 1C), indicating that a loss of function of mp29 might be embryonic lethal.

### Abnormality of *mp29^GT/GT^* embryos

**Figure 2:**
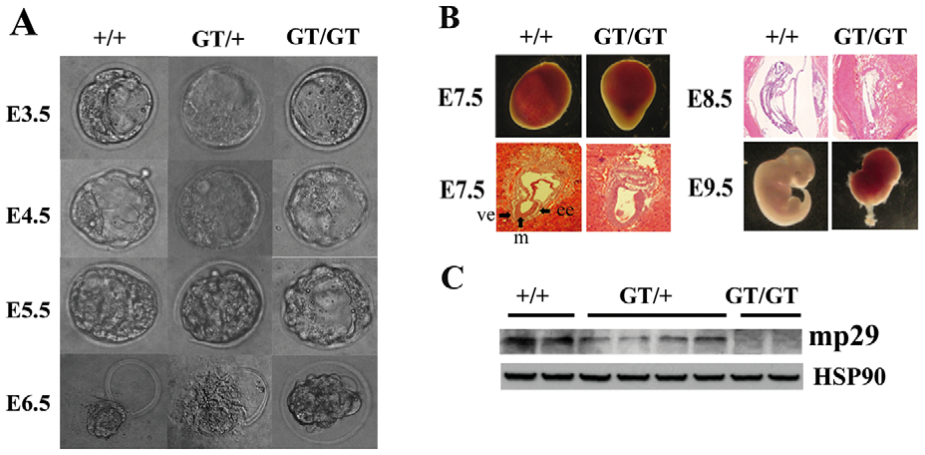
Loss of *mp29* resulted in aberrant embryonic development. The following results were repeated with three independent experiments. (A) Morphology of embryos cultured *in vitro*. Embryos were collected from uterus at E3.5 and cultured *in vitro* to E6.5. Embryos were hatched at E6.5 in *mp29^+/+^* and *mp29^GT/+^* embryos. (B) Embryos were isolated from E7.5 to E9.5 for hematoxylin–eosin staining. *mp29^+/+^* and *mp29^GT/+^* embryos at E7.5 had normal gastrulation. ee: embryonic ectoderm. m: mesoderm. ve: visceral endoderm. (C) Whole embryo extracts at E7.5 were lysed in RIPA buffer for Western blotting analysis. Hsp90 was used as a loading control.

E3.5 blastocysts were collected and cultured individually *in vitro*. Genotyping of embryos was carried out using PCR analysis (Figure S2 for the second supporting information figure). We did not find any *mp29^GT/GT^* embryos after E11.5 (Table 1). Microscopic inspection of each embryo reveals no gross morphologic defects from E3.5 to E5.5 (Fig. 2A and Figure S2B for the second supporting information figure). In *mp29^+/+^* blastocysts, normal trophoblast giant cells and inner cell mass could be observed at E6.5. However, *mp29^GT/GT^* blastocysts exhibited a shrink shape without hatching (Fig. 2A).

**Table 1:** Analysis of embryos from intercrosses of *mp29^GT/+^* mice.

			Genotypes		
Stages	Embryos	+/+	GT/+	GT/GT	Abnormality
E13.5	7	3	4	0	0
E11.5	7	2	5	0	0
E9.5	21	5	11	5	5
E8.5	18	4	10	4	4
E7.5	20	5	11	4	4
E6.5	18	4	10	4	4
E5.5	18	4	10	4	0
E4.5	18	4	10	4	0

**Figure 3:**
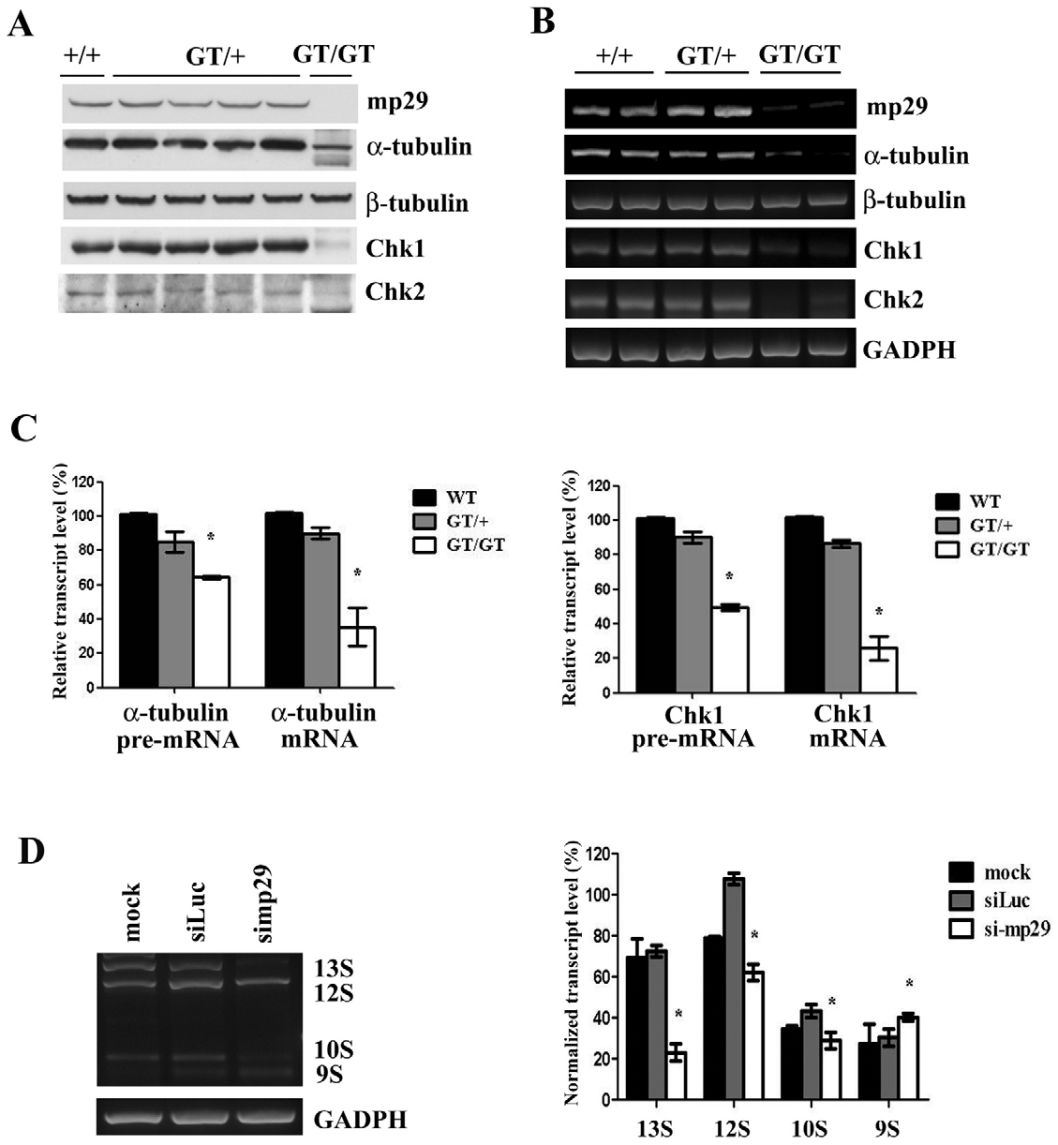
Decreased α-tubulin and Chk1 expression in *mp29^GT/GT^* embryo. Unless otherwise stated, the following results were repeated with three independent experiments. (A) Whole embryo extracts at E7.5 were prepared for Western blotting analysis. (B) RT-PCR for embryos prepared from E7.5. Note lower levels of α-tubulin, Chk1, and Chk2, but not β-tubulin, in *mp29^GT/GT^* embryos. GAPDH was used as an input control. (C) Total RNAs from embryos at E7.5 were extracted and first strand cDNAs were transcribed for quantitative real-time PCR analysis to determine the relative pre-mRNA and mRNA levels of α-tubulin and Chk1. GAPDH was used as a normalized control. (D) NIH3T3 cells were co-transfected with siRNAs and E1A splicing reporter for *in vivo* alternative splicing assay. Total RNAs were isolated and subjected to RT-PCR. The splicing products were quantitatively analyzed with three independent experiments. GAPDH was used as a normalized control. * indicated *p*<0.05 in one-way ANOVA F-test.

At E7.5, *mp29^GT/GT^* embryos did not undergo normal gastrulation, which led to the formation of embryonic ectoderm, mesoderm, and endoderm (Fig. 2B). Furthermore, *mp29^GT/GT^* embryos at E8.5. and E9.5 displayed a severely impaired embryonic development of head, trunk, and appendages. In contrast, *mp29^+/+^ mp29^GT/+^* embryos at E8.5 had normal organogenesis (Fig. 2B). Western blot analysis showed the absence of mp29 protein in *mp29^GT/GT^* embryos at E7.5 (Fig. 2C), suggesting that the ablation of mp29 deteriorated the survival of *mp29^GT/GT^* embryos. A low level of mp29 appeared after a long exposure for immunoblotting or RT-PCR analyses (Fig. 2C and Fig. 3B), which was likely due to an incomplete interruption of inserted *mp29* gene or a contamination of mother tissues.

### Reduced α-tubulin and Chk1 expression in *mp29^GT/GT^* embryos

Previous studies show that double mutant *isy1Δ and syf2Δ* lead to a low level of α-tubulin expression in yeast and p29 affects Chk1 and Chk2 expression in human cells [3], [7]. Thus, we examined the expression of α-tubulin, Chk1 and Chk2 in *mp29^GT/GT^* embryos. As shown in Fig. 3A, the protein levels of α-tubulin, Chk1, and Chk2 were reduced in *mp29^GT/GT^* embryos at E7.5. RT-PCR revealed decreased mRNA levels of α-tubulin, Chk1, and Chk2 (Fig. 3B). Quantitative real-time PCR analysis showed lower levels of α-tubulin and Chk1 pre-mRNA and mRNA transcripts in *mp29^GT/GT^* embryos at E7.5 compared with wild-type and *mp29^GT/+^* embryos (Fig. 3C), indicating that mp29 might be involved in transcriptional and post-transcriptional regulation of α-tubulin and Chk1. RNA immunoprecipitation showed that HA-mp29 preferentially pulled down pre-mRNAs of α-tubulin and Chk1 in NIH3T3 cells, but only a much smaller amounts of β-tubulin and Chk2 pre-mRNAs were pulled down (Figure S3 for the third supporting information figure), indicating that the decreased Chk2 expression in *mp29^GT/GT^* embryos might not be a direct effect of mp29 deficiency.

The isolation of mouse embryonic fibroblasts (MEFs) from *mp29^GT/GT^* embryos at E7.5 was unsuccessful, possibly due to its lack of viability. Alternatively, we silenced mp29 expression in mouse NIH3T3 embryonic fibroblasts using RNA interference strategy. Introduction of mp29 siRNA duplexes into NIH3T3 cells significantly reduced the mRNA and protein levels of α-tubulin and Chk1, but had a minimal effect on those of β-tubulin and Chk2 (Fig. 3 and Figure S3A for the third supporting information figure). Additionally, after transfection of an E1A alternative splicing reporter into NIH3T3 cells, mp29 depletion significantly decreased expression levels of 13S, 12S, and 10S transcripts with an elevated 9S transcript expression (Fig. 3D), suggesting that the mp29 might play a role in the post transcriptional control on its target genes both in *mp29^GT/GT^* embryos and mp29 silenced NIH3T3 cells.

### Defective checkpoint in *mp29^GT/GT^* embryos

**Figure 4:**
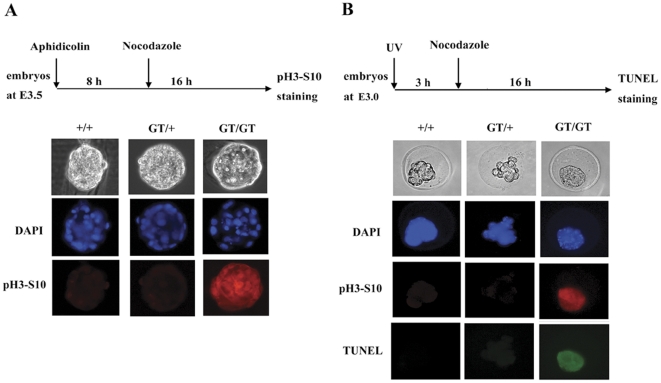
Impaired G2/M checkpoint in *mp29^GT/GT^* embryos. Unless otherwise stated, the following results were repeated with three independent experiments. (A) *mp29^+/+^*, *mp29^GT/+^*, and *mp29^GT/GT^* blastocysts at E3.5 were treated with aphidicolin (1 µM) for 8 h, incubated with nocodazole (0.1 µg/ml) for an additional 16 h, and then fixed and stained with antibody specific to phosphohistone H3 at Ser10. (B) *mp29^+/+^*, *mp29^GT/+^*, and *mp29^GT/GT^* blastocysts at E3.0 were irradiated with UV light (30 J/m^2^), incubated with nocodazole for 16 h, and then co-immunostained with anti-phosphohistone H3 at Ser10 antibody and TUNEL fluorescein. The genotypes of each embryo were determined by PCR. Images were obtained using Leica DM6000B microscope.

In accordance with our previous results [7], mouse Chk1 phosphorylation at S345 was reduced in mp29 knockdown NIH3T3 cells upon UV irradiation (Figure S3B for the third supporting information figure). To examine the effects of mp29 deficiency on DNA damage response in *mp29^GT/GT^* embryos, we collected E3.5 blastocysts from heterozygous intercross and cultured these embryos individually in the presence of aphidicolin to induce DNA replication stress, followed by nocodazole treatment for cell cycle arrest at the prophase. The immunostaining of anti-Histone H3 phosphorylated at Ser10, an M phase specific marker, showed that phosphohistone H3-positive cells were significantly increased in aphidicolin and nocodazole treated *mp29^GT/GT^* embryos (Fig. 4A). Similarly, phosphohistone H3-positive cells were increased in *mp29^GT/GT^* embryos and terminal deoxytransferase-mediated deoxyuridine nick end-labeling (TUNEL)-positive cells were detected in *mp29^GT/GT^* embryos at E3.0 after UV light exposure and nocodazole treatment (Fig. 4B). Additionally, *mp29^GT/GT^* embryos irradiated with UV light exhibited an increased level of γ-H2AX staining (Figure S4 for the fourth supporting information figure). Together, these results indicated an insufficient checkpoint capacity at G2/M to arrest cell cycle progression in *mp29^GT/GT^* embryos, which might be responsible for the genome instability and the restrictive viability upon replication stress and DNA damage.

### Complementation of mp29 expression with *mp29* transgenic mice

**Figure 5:**
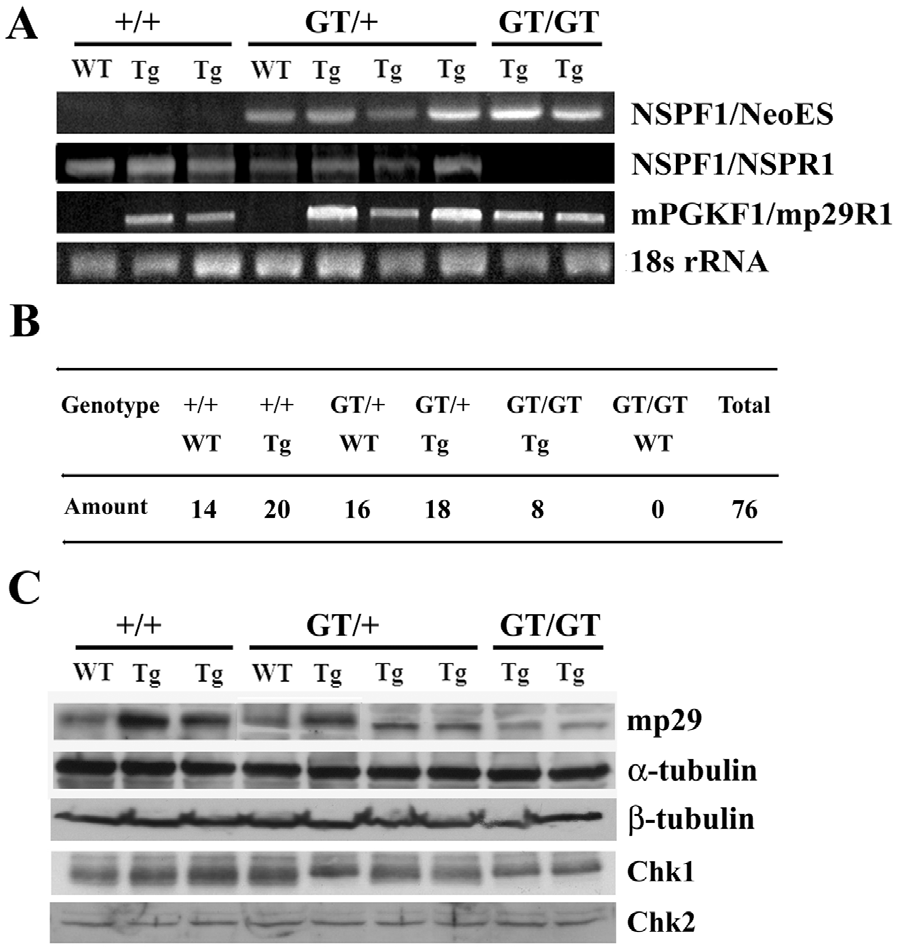
*mp29* transgene complemented the deficiency of mp29. *mp29^GT/+^* mice were mated with *mp29^Tg/+^* mice to generate *mp29^GT/+^mp29^Tg/+^* littermates. Inbreed of *mp29^GT/+^mp29^Tg/+^* male mice with *mp29^GT/+^mp29^+/+^* female mice gave birth to *mp29^GT/GT^mp29^Tg/+^* mice. (A) Genotyping of complemented mice by PCR analysis. NSPF1/NeoES PCR products indicated the presence of U3NeoSV1 vector. NSPF1/NSPR1 primers were used to determine whether both of alleles were inserted with U3NeoSV1 vector. mPGKF1/mp29R1 PCR products indicated the presence of *mp29* transgene with a product of 500 bp. Tg refers to the hemizygous *mp29* transgene. (B) The genotyping of 76 complemented offsprings was examined. Eight pups exhibited homozygously interrupted genotype but with mp29 transgene (*mp29^GT/GT^mp29^Tg/+^*). There were no *mp29^GT/GT^* mice that could survive without the presence of *mp29* transgene. (C) Tissue extracts of the livers from complemented mice were prepared for Western blot analysis. Note that normal expression levels of α-tubulin, Chk1, and Chk2 were restored in *mp29^GT/GT^mp29^Tg/+^* mice.

To avert the possibility that *mp29^GT/GT^* abnormality resulted from a random integration by the gene trap vector, *mp29^GT/+^mp29^Tg/+^* male littermates were generated by outcrossing *mp29^GT/+^* mice in the 129Sv2/C57BL6 mixed background with *mp29^Tg/+^* transgenic mice in the FVB genetic background. Inbreeding of *mp29^GT/+^mp29^Tg/+^* male mice with *mp29^GT/+^mp29^+/+^* female mice gave birth to *mp29^GT/GT^mp29^Tg/+^* mice. Genotyping results revealed the presence of *mp29* transgene in eight homozygous *mp29* gene-trap mice among 76 offsprings. As anticipated, no embryonic lethality occurred in these *mp29^GT/GT^mp29^Tg/+^* mice (Fig. 5A and 5B). The expression of mp29, α-tubulin, Chk1, and Chk2 were restored in *mp29^GT/GT^mp29^Tg/+^* mice (Fig. 5C). Morphology of *mp29^GT/GT^mp29^Tg/+^* mice is indistinguishable from *mp29^+/+^mp29^Tg/+^* and *mp29^GT/+^mp29^Tg/+^* mice. Furthermore, histological staining of liver and spleen tissues from *mp29^GT/GT^mp29^Tg/+^* mice did not show significant differences from those of *mp29^+/+^mp29^Tg/+^* and *mp29^GT/+^mp29^Tg/+^* mice (Figure S5 for the fifth supporting information figure). Collectively, the molecular and morphological defects in *mp29^GT/GT^* mice could be rescued by *mp29* transgene.

### Morpholino knockdown of *zfp29* in zebrafish

**Figure 6:**
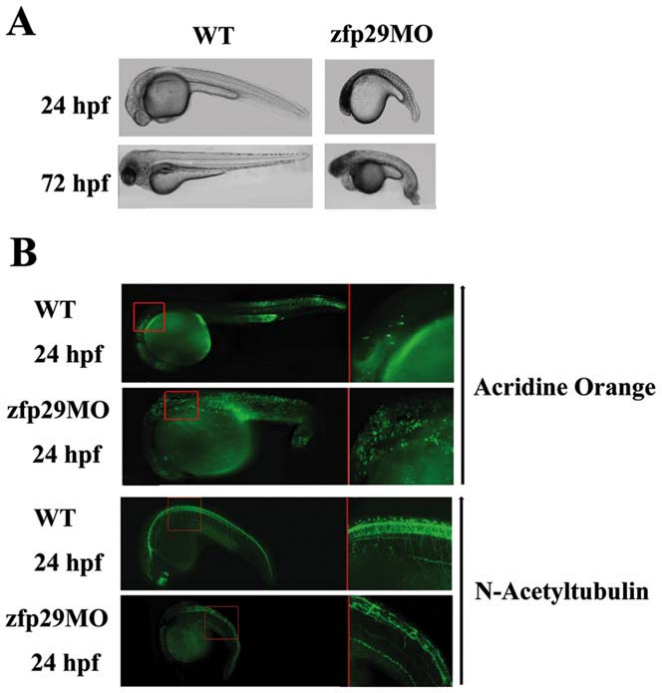
Effects of MO-mediated zfp29 knockdown in Zebrafish. One hundred zebrafish embryos at 2-cell stage were injected with control and zfp29MO. 76 embryos had phenotypic defects. (A) Lateral view of live embryos at 24 hpf. (B) Embryos were injected with control and zfp29 MO and then incubated with acridine orange or incubated with anti-acetylated tubulin antibody at 24 hpf. Increased apoptotic cells were shown with acridic orange (AO) in zfp29 depleted cells. Reduction of acetylated α-tubulin and less motor neurites were identified in zfp29 morphants.

Amino acid alignment showed that mouse mp29 shares nearly 80% sequence identify with zebrafish zfp29 and 12.3% sequence identity with yeast Syf2 (Figure S1 for the first supporting information figure). Since the amino acid sequences of p29 were highly conserved in vertebrates, we examined whether zfp29 deficiency would also result in an impaired embryogenesis. One hundred zebrafish embryos at 2–4 cells were injected with a zfp29 morpholino oligonucleotide, zfp29MO. The morphological results showed that more than 75% of the zfp29 morphants exhibited an abnormal development, including a small head, yolk extension, bent trunk, and crooked tail, at 24 hpf and died at 72 hpf (Fig. 6A). Acridine orange staining, a fluorescent dye to identify engulfed apoptotic cells, indicated a remarkable increase in apoptotic cells in zfp29MO-injected embryos. Since there were no suitable antibodies against Chk1 in zebrafish, we checked the expression level of acetylated α-tubulin, an early developmental marker for motor neurons. The distribution of acetylated α-tubulin was significantly reduced and less neurons/neurites appeared in zfp29 morphants compared with uninjected embryos (Fig. 6B). To further support these neuronal deformities, a wholemount *in situ* RNA hybridization against *HuC*, an early pan-neuronal marker in zebrafish [11], also displayed a significant loss of neuronal cells in the spinal cord in zfp29 morphants. Additionally, knockdown of zfp29 in the *Fli* transgenic line [12], which specifically expresses green fluorescence protein in blood vessels, also disturbed the blood vessel formation (Figure S6 for the sixth supporting information figure). Taken together, these results indicated that the abnormities of zfp29 knockdown affected the whole body development and were not restricted to specific tissues.

## Discussion

We have generated mp29 knockout mice from the *mp29* gene-trap embryonic stem cell. Mice carrying a heterozygous insertion between exon 2 and exon 3 of *mp29* gene were viable and their offsprings are able to inherit the mutated allele. However, embryos inherited with homozygous insertion in *mp29* gene were not viable and *mp29^GT/GT^* embryos showed developmental defects at E6.5, partly due to an aberrant DNA damage checkpoint in cell cycle.

Mass spectrometry analysis identified several major components for pre-mRNA splicing, such as U5 snRNP, Prp 8, and hnRNPA2/U, in HA-mp29 immunoprecipitated complexes, suggesting an association of mp29 with pre-mRNA complex (Table S1 for the first supporting information Table). Homozygous inactivation of mp29/Syf2 in mice embryos and knockdown of mp29 in NIH-3T3 cells profoundly decrease the expression of α-tubulin and Chk1. In contrast, no significant effect was on β-tubulin and Chk2 (Fig. 3 and Figure S3 for the third supporting information figure). This result is contrary to the deletion of *SYF2* from the previous study in *Saccharomyces cerevisiae*, which has no notable difference cell cycle progression or in splicing of U3 transcripts. Instead, a lower expression of α-tubulin was only found in double mutant *isyl1Δ and syf2Δ* cells [3], [4]. This conflicted phenomenon can be explained by the low similarity between mouse mp29 and yeast Syf2. There might be different regulatory mechanisms that prevail in these two species. This contention is also supported by the decreased expression of acetylated α-tubulin in zfp29 depleted zebrafish (Fig. 6B). However, knockdown of human p29 in HeLa and U2OS does not result in an alleviated α-tubulin expression [7], indicating that depletion of human p29 may not affect the post-transcriptional regulation of α-tubulin in human cancer cells.

Several DDR- and pre-mRNA splicing-related knockout mice have been investigated. For example, *ATM* knockout mice are viable, but display growth retardation, infertility, defects in T lymphocyte maturation, and extreme sensitivity to γ-irradiation [13]. *Chk2*-deficient mice exhibit radioresistance and defective p53-mediated transcription [14]. By contrast, the absence of main players in replication checkpoint results in severely developmental abnormalities. Aberrant cell cycle checkpoint and embryonic death as early as E3.5 are found in *ATR* and *Chk1* deficient mice [15]–[17], implying that *ATR* and *Chk1* are essential for the embryonic development and distinct from *ATM* and *Chk2*. Although major events associated with DNA damage and replication checkpoints are well defined, it is unclear whether there is a splicing checkpoint to prevent errors during pre-mRNA splicing process. Several components of splicing factors have been identified to participate in pre-mRNA splicing and DNA repair. For instance, SNEV (Prp19/Pso4) is a nuclear matrix protein involved in pre-mRNA splicing, ubiquitylation, and DNA repair. Mouse *SNEV* is indispensable for early mouse development and mutant *SNEV* results in preimplantation lethality at E3.5 [18]. The mammalian Pso4 complex, PSO4/PRP19/SNEV, CDC5L, PLRG1, and SPF27, is involved in both pre-mRNA splicing and DNA damage response [19]. Cdc5L interacts with ATR and is involved in the regulation of the ATR-mediated cell-cycle checkpoint in response to genotoxic agents [20]. Inactivation of PLRG1 in mice results in embryonic lethality at 1.5 days post-fertilization [21]. Disruption of murine *mp29* gene resulted in the embryonic lethality, which is reminiscent of the phenotype of *ATR*, *Chk1*, *SNEV*, and *PLRG1*, leading to a conclusion that mp29/Syf2/Ntc31 may play substantial roles in DNA damage response by modulating transcriptional and post-transcriptional control of a certain set of target pre-mRNAs.

## Materials and Methods

### Animals

A gene trapped mouse E14 embryonic stem cell (PST25562-NR) with a 129Sv2 background was obtained from Mammalian Functional Genomics Center [22]. The E14 ES cell was injected into mouse strain C57BL/6 blastocysts to generate chimeric mice by Transgenic Core Facility at the National Taiwan University Hospital. C57BL/6 mice were purchased from BioLASCO Taiwan. The establishment of mp29 transgenic FVB mice has been described [9]. We would be willing to provide the mice models for the research purpose upon request.

### Ethics statement

All animal studies were performed in compliance with the protocol #97060 of the Institutional Animal Use and Care Committee, National Taiwan University. All efforts were carried out to minimize suffering.

### Generation of mp29 knockout mice

Two independent male chimeric mice with germ line transmission were back-crossed with female C57BL/6 mice to generate mp29 heterozygous F1 offsprings. Heterozygous male and female mice were bred to isolate wild-type (*mp29^+/+^*), heterozygous (*mp29^GT/+^*), and homozygous mutant (*mp29^GT/GT^*) mice.

### Genotyping

Mouse tails (0.5 cm) were cut with scissors, ground to homogenize the tail tissues, and incubated in 20 µl of Proteinase K (10 mg/ml) at 60°C for 30 min. Tail DNAs were isolated using EasyPure Genomic DNA spin kit (Bioman, Taipei, Taiwan) for PCR. The following pairs of primers were used to examine the presence of inserting vector, U3NeoSV1. NSPF1: 5′-CATTACCGGGTGTACCTTAGGT-3′; NeoES 5′-AATCCATCTTGTTCAATCATGC-3′ and Ltr 5′-AGTTGCATCCGACTTGTG-3′; NSPR1 5′-GCATGCC TTTTGAATTATAGTCC-3′. NSPF1 and NSPR1 were used to amplify the untargeted *mp29* allele with a product of 300 bp, which would be absent in *mp29^GT/GT^* mutants. PCR conditions were performed at 95°C for 3 min followed by 35 cycles of 95°C for 20 s, 57°C for 20 s, and 72°C for 30 s.

### Blastocyst culture

Embryos at E3.5 were collected by flushing the uterus with PBS and cultured in HEPES-buffered M2 medium (Sigma-Aldrich) for 4 days until embryos hatched. Embryos were inspected daily and photographed to monitor their developments.

### Western blotting

Protein extracts from mouse NIH3T3 cells, embryos, and adult tissues were solubilized in RIPA buffer (25 mM Tris-HCl pH 7.5, 150 mM NaCl, 1% NP-40, 1% sodium deoxycholate, 0.1% SDS, protease inhibitors) on ice for 30 min and sonicated for 5 min using a sonicator Misonix S-4000 (Farmingdale, NY). Rabbit anti-Chk1 and Chk2 antibodies were purchased from Cell Signaling (Beverly, MA). Mouse anti-α/β-tubulin and acetylated α-tubulin antibodies were from Sigma-Aldrich (St. Louis, MI). Mouse anti-GAPDH antibody was obtained from Santa Cruz Biotech (Santa Cruz, CA). Mouse anti-mp29 monoclonal antibody has been described [10].

### RNA isolation and quantitative real time PCR

Total RNA was isolated using Trizol, treated with RNase-free DNase I (Promega), and transcribed into cDNA using Superscript III reverse transcriptase (Invitrogen). Real time quantitative PCR was performed using the Maxima SYBR Green kit (Fermentas, CA) on a ABI 7300 Sequence Detection System (PE Applied Biosystems) using a heat-activated TaqDNA polymerase (PE Applied Biosystems). The sequences of primers were listed below: for α-tubulin pre-mRNA (5′-TGAGCCAGCCAACCAGATG-3′ and 5′-TGGTCTTGATGGTGGCAATG-3′), for α-tubulin mRNA (5′-CCAGCCCCCCAGGTTTC-3′ and 5′-TGTCTACCATGAAGGCACAATCA -3′), for Chk1 pre-mRNA (5′-TATCCCCACCCCTCAGTTTTG-3′ and 5′-AGAGACACCTCCACCCGCT-3′), for Chk1 mRNA (5′-CTGGTTCAGGGCATCAGTTTTT-3′ and 5′-GGTAAGATTTGTCCGCATCCA-3′), for GAPDH (5′-TGACATCAAGAAGGTGGTGAAG-3′ and 5′-AGAGTGGGAGTTGCTGTTGAAG-3′). The relative transcript amount of the target gene, which was calculated using standard curves of serial RNA dilutions, was normalized to that of GAPDH of the same RNA.

### siRNA transfection

An mp29 siRNA, 5′-CAGAGGAAAUUGACAGAA-3′, was incubated with RNAiMax lipofectamine (Invitrogen) at room temp for 20 min. Cells were washed with PBS and incubated in serum-free culture medium. The siRNA-RNAiMax complex was dropped onto cells and mixed gently by rocking the plate back and forth. Fetal bovine serum was added at 8 h post-transfection. Cell extracts were collected at 72 h post-transfection.

### *In vivo* splicing assay

NIH3T3 cells were transfected with the E1A expression vector (pCEP4-E1A) as previously described [23] with minor modifications. Briefly, NIH3T3 cells were transfected with 20 nM of siRNA for 48 h and then co-transfected with E1A vector for another 24 h. Total RNA was isolated using Trizol reagent and treated with RQ-DNase I. RT-PCR was performed using primer P1 (5′-GGTCTTGCAGGCTCCGGTTCTGGC-3′) and P2 (5′-GCAAGCTTGAGTGCCAGCGAGTAG-3′). PCR products were fractionated on agarose gels and images were recorded. Quantitative results were carried out with three independent experiments.

### Immunofluorescence

Embryos were fixed with 4% paraformaldehyde in PBS for 20 min and then permeabilized for 10 min at room temperature with PBS containing 0.3% Triton X-100. The aphidicolin or UV treated embryos were stained with DAPI and labeled with antibody specific to phosphohistone H3 at Ser10 (Cell Signaling). DNA fragmentation associated with apoptosis was detected with an *in situ* cell death detection kit (Roche). Permeabilized embryos were double stained with anti-phosphohistone H3 at Ser10 antibody and TUNEL reaction mixture for 60 min at 37°C. Fluorescein-labeled DNA was analyzed using a fluorescence microscope.

### Complementation of mp29 deficiency with mp29 transgenic mice

*mp29^GT/+^* mice were outcrossed with *mp29^Tg/+^* mice to produce *mp29^GT/+^mp29^Tg/+^* littermates. Inbreeding of *mp29^GT/+^mp29^Tg/+^* male mice with *mp29^GT/+^mp29^+/+^* female mice gave birth to *mp29^GT/GT^mp29^Tg/+^* mice. Genotyping was performed as described above.

### Morpholino injection

Respective morpholino oligonucleotides (MOs) were synthesized by Gene Tools (Philomath, OR, USA). The MO was dissolved in Danieau solution containing 0.5% phenol red and 3.2 ng per zebrafish embryo (*Danio rerio*) was injected into embryos at the two-cell stage. MO sequences are: zfmp29, 5′-TCGCTAGACGCCATGTTGCTTTTCG-3′. After 24 hpf, apoptotic cells were stained with acridine orange (Sigma-Aldrich, St. Louis, MO). Injected and uninjected embryos were fixed and immunostained with anti-acetylated α-tubulin antibody (Sigma-Aldrich).

## Supporting Information

Figure S1 **Amino acid alignment of human p29 (AAG42073), mouse mp29 (NP_081058), zebrafish zfp29 (NP_001003437), and yeast Syf2 (NP_011645).**
(TIF)

Figure S2 **Genotyping and histological analyses of** ***mp29*** **gene-trap embryos.** (A) PCR analysis was conducted to determine the genotypes of embryos at E6.5. NSPF1/NeoES were used to detect the presence of gene trap vector and NSPF1/NSPR1 were used to identify homozygotes with two *mp29* interrupted alleles. 18srRNA was used as a control. (B) Histological analysis of *mp29* gene-trap embryos at E5.5.
(TIF)

Figure S3 **RNA immunoprecipitation and siRNA depletion in NIH3T3 cells.** (A) Mouse NIH3T3 cells were transfected with empty HA vector or HA-mp29 for 48 h and immunoprecipitated by anti-HA agarose. Total RNAs were transcribed for RT-PCR for indicated targets. (B) Mouse NIH3T3 cells were transfected with siRNAs for 72 h and total RNAs were transcribed for RT-PCR analysis. (C) NIH3T3 cells were transfected with siRNA duplexes for 72 h and whole cell extracts were prepared for Western blot analysis. (D) Mouse NIH3T3 cells were transfected with siRNA duplexes for 72 hours and then irradiated with UV light (50 J/m^2^). Cell extracts were harvested at 3 h post-UV treatment and Western blot was carried out using anti-Chk1 and Chk1 S345 antibodies. Note that a decrease of Chk1 phosphorylation at S345 in mp29 depleted cells. Hsp90 was used as a loading control.
(TIF)

Figure S4 **Detection of γH2AX in mp29 gene-trap embryos.** *mp29^+/+^*, *mp29^GT/+^*, and *mp29^GT/GT^* blastocysts at E3.5 were irradiated with UV light (50 J/m^2^), and then immunostained with anti-γH2AX antibody. The genotypes of each embryo were determined by PCR. Images were obtained using Leica DM6000B microscope.
(TIF)

Figure S5 **Macro-inspection of** ***mp29*** **transgene complement mice.** (A) *mp29^GT/+^* mice were outcrossed with *mp29^Tg/+^* mice to generate *mp29^GT/+^mp29^Tg/+^* littermates. Inbreeding of *mp29^GT/+^mp29^Tg/+^* with *mp29^GT/+^mp29^+/+^* mice gave birth to *mp29^GT/GT^mp29^Tg/+^* mice. Morphology of *mp29^GT/+^mp29^Tg/+^* inbreeding mice. (B) Hematoxylin–eosin staining of the liver and spleen tissues isolated from *mp29^+/+^mp29^Tg/+^*, *mp29^GT/+^mp29^Tg/+^*, and *mp29^GT/GT^mp29^Tg/+^* mice.
(TIF)

Figure S6 **Expression of HuC and blood vessel formation in zfp29 knockdown morphants.** (A) Zebrafish embryos at 2-cell stage were uninjected or injected with zfp29 MO and then probed with DIG-labeled HuC probe for in situ hybridization at 24 hpf. (B) Two-cell embryos of *Fli* transgenic line were injected with control and zfp29 MO and then fixed for detection of green fluorescence protein expression.
(TIF)

Table S1 **List of mp29 associated proteins identified by Mass spectrometry analysis.**
(DOCX)
