# Supplementary figures and images for: Disruption of Murine mp29/Syf2/Ntc31 Gene Results in Embryonic Lethality with Aberrant Checkpoint Response
Source: PLoS One. 2012 Mar 20;7(3):e33538. doi: 10.1371/journal.pone.0033538 (PMC3308990; doi:10.1371/journal.pone.0033538)

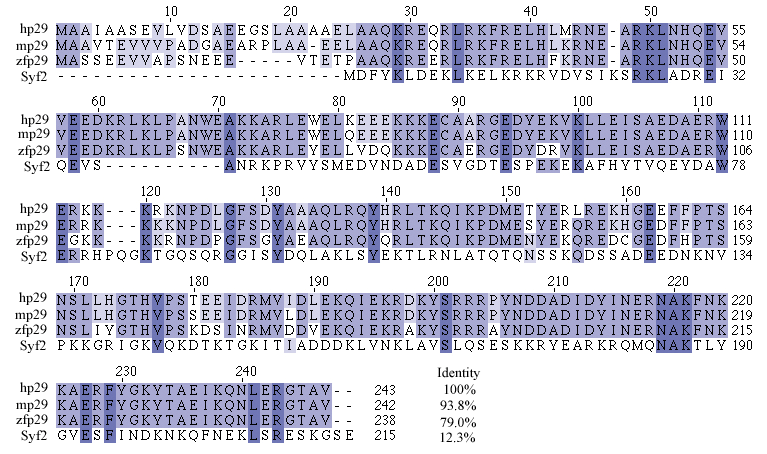

Supplement: Figure S1 — Amino acid alignment of human p29 (AAG42073), mouse mp29 (NP_081058), zebrafish zfp29 (NP_001003437), and yeast Syf2 (NP_011645). (TIF) [file pone.0033538.s001.tif]

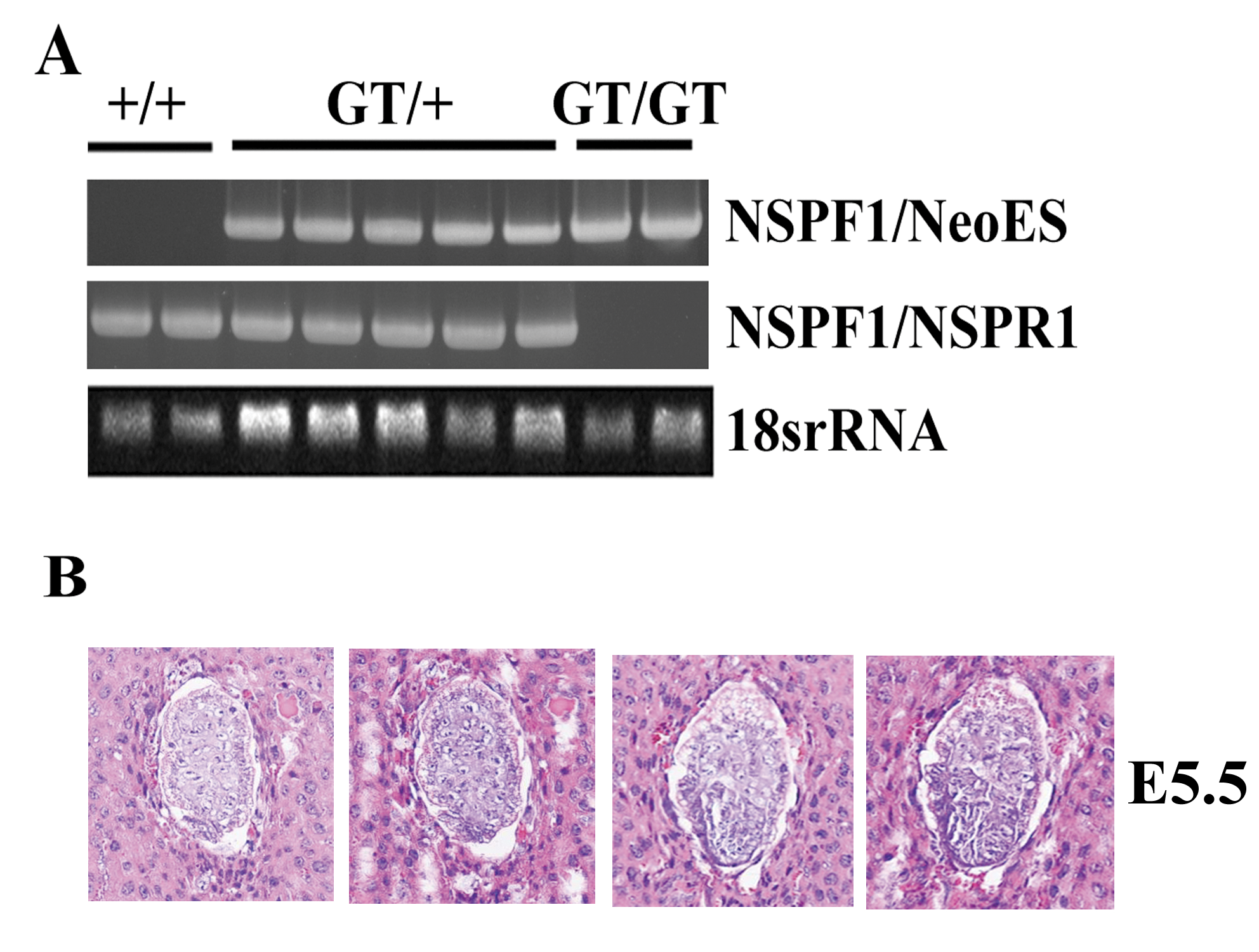

Supplement: Figure S2 — Genotyping and histological analyses of mp29 gene-trap embryos. (A) PCR analysis was conducted to determine the genotypes of embryos at E6.5. NSPF1/NeoES were used to detect the presence of gene trap vector and NSPF1/NSPR1 were used to identify homozygotes with two mp29 interrupted alleles. 18srRNA was used as a control. (B) Histological analysis of mp29 gene-trap embryos at E5.5. (TIF) [file pone.0033538.s002.tif]

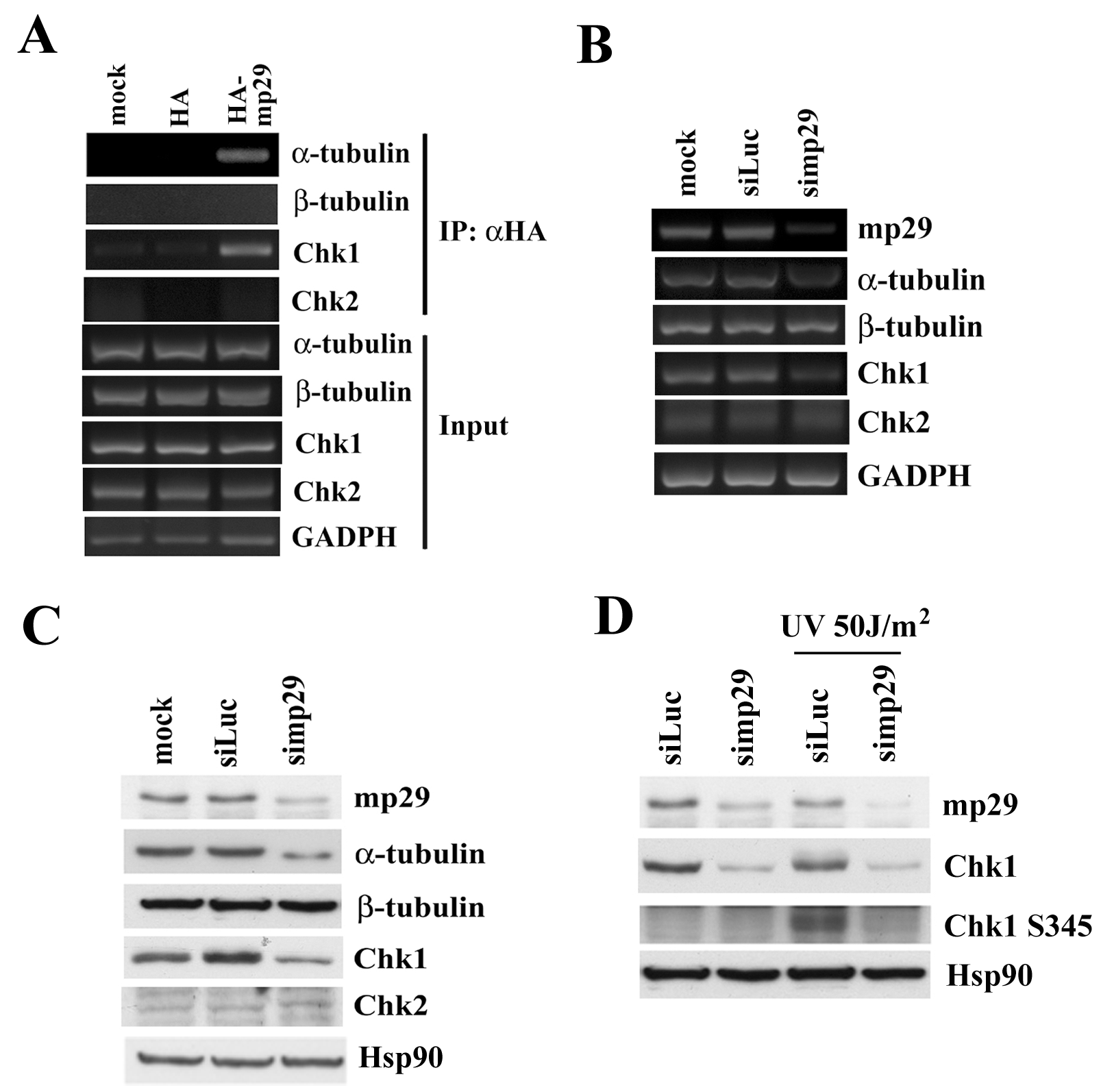

Supplement: Figure S3 — RNA immunoprecipitation and siRNA depletion in NIH3T3 cells. (A) Mouse NIH3T3 cells were transfected with empty HA vector or HA-mp29 for 48 h and immunoprecipitated by anti-HA agarose. Total RNAs were transcribed for RT-PCR for indicated targets. (B) Mouse NIH3T3 cells were transfected with siRNAs for 72 h and total RNAs were transcribed for RT-PCR analysis. (C) NIH3T3 cells were transfected with siRNA duplexes for 72 h and whole cell extracts were prepared for Western blot analysis. (D) Mouse NIH3T3 cells were transfected with siRNA duplexes for 72 hours and then irradiated with UV light (50 J/m2). Cell extracts were harvested at 3 h post-UV treatment and Western blot was carried out using anti-Chk1 and Chk1 S345 antibodies. Note that a decrease of Chk1 phosphorylation at S345 in mp29 depleted cells. Hsp90 was used as a loading control. (TIF) [file pone.0033538.s003.tif]

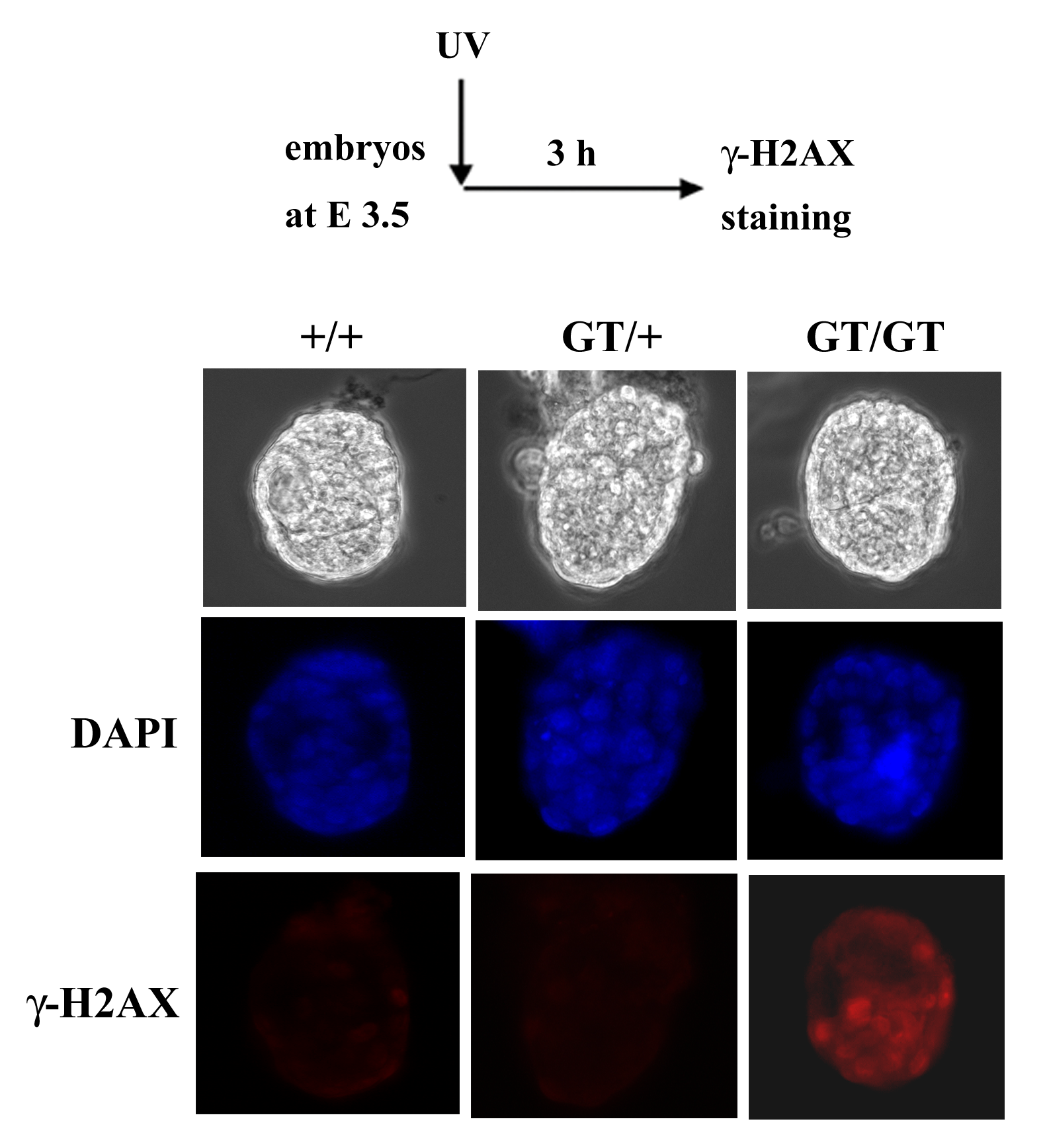

Supplement: Figure S4 — Detection of γH2AX in mp29 gene-trap embryos. mp29+/+, mp29GT/+, and mp29GT/GT blastocysts at E3.5 were irradiated with UV light (50 J/m2), and then immunostained with anti-γH2AX antibody. The genotypes of each embryo were determined by PCR. Images were obtained using Leica DM6000B microscope. (TIF) [file pone.0033538.s004.tif]

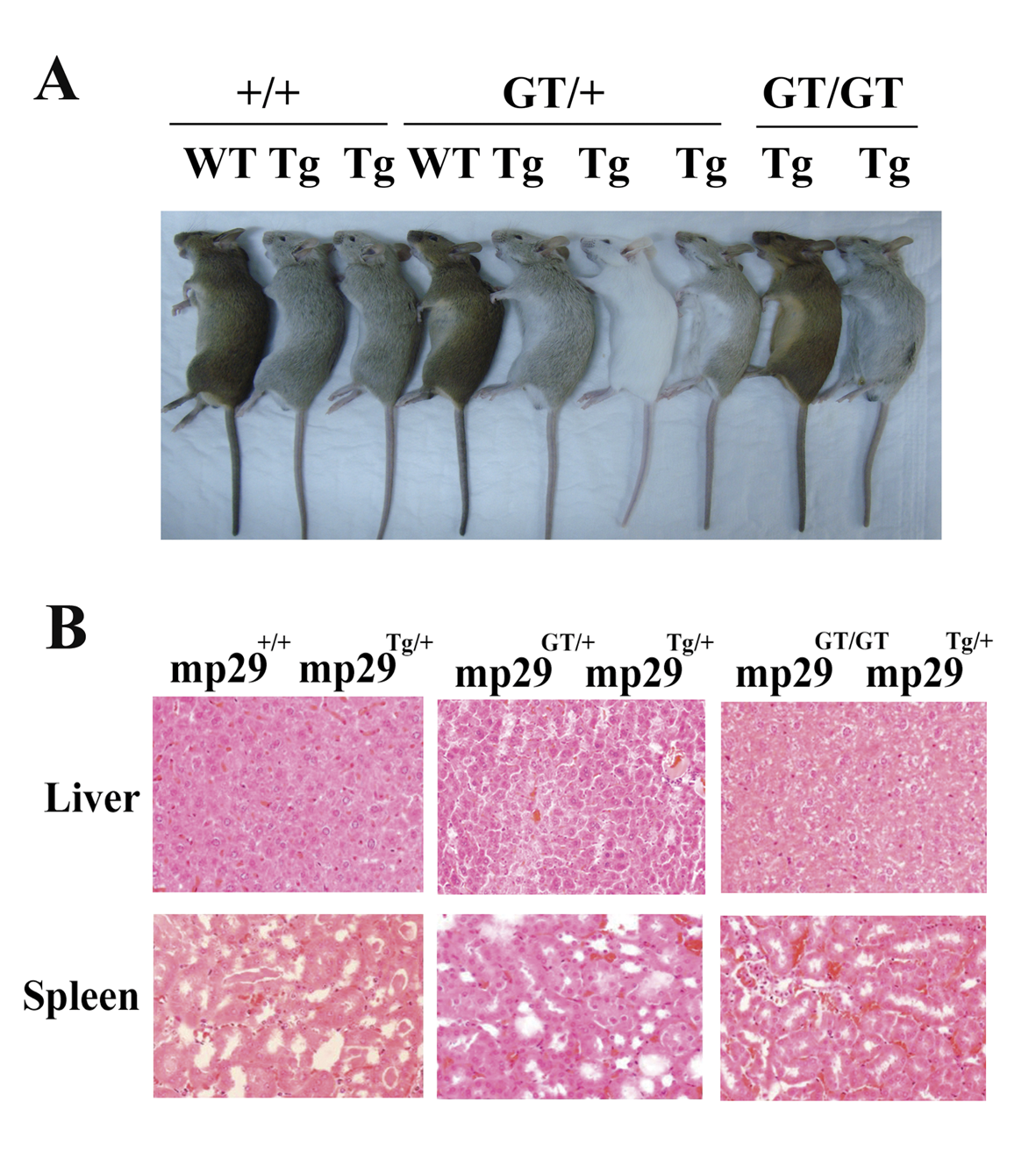

Supplement: Figure S5 — Macro-inspection of mp29 transgene complement mice. (A) mp29GT/+ mice were outcrossed with mp29Tg/+ mice to generate mp29GT/+mp29Tg/+ littermates. Inbreeding of mp29GT/+mp29Tg/+ with mp29GT/+mp29+/+ mice gave birth to mp29GT/GTmp29Tg/+ mice. Morphology of mp29GT/+mp29Tg/+ inbreeding mice. (B) Hematoxylin–eosin staining of the liver and spleen tissues isolated from mp29+/+mp29Tg/+, mp29GT/+mp29Tg/+, and mp29GT/GTmp29Tg/+ mice. (TIF) [file pone.0033538.s005.tif]

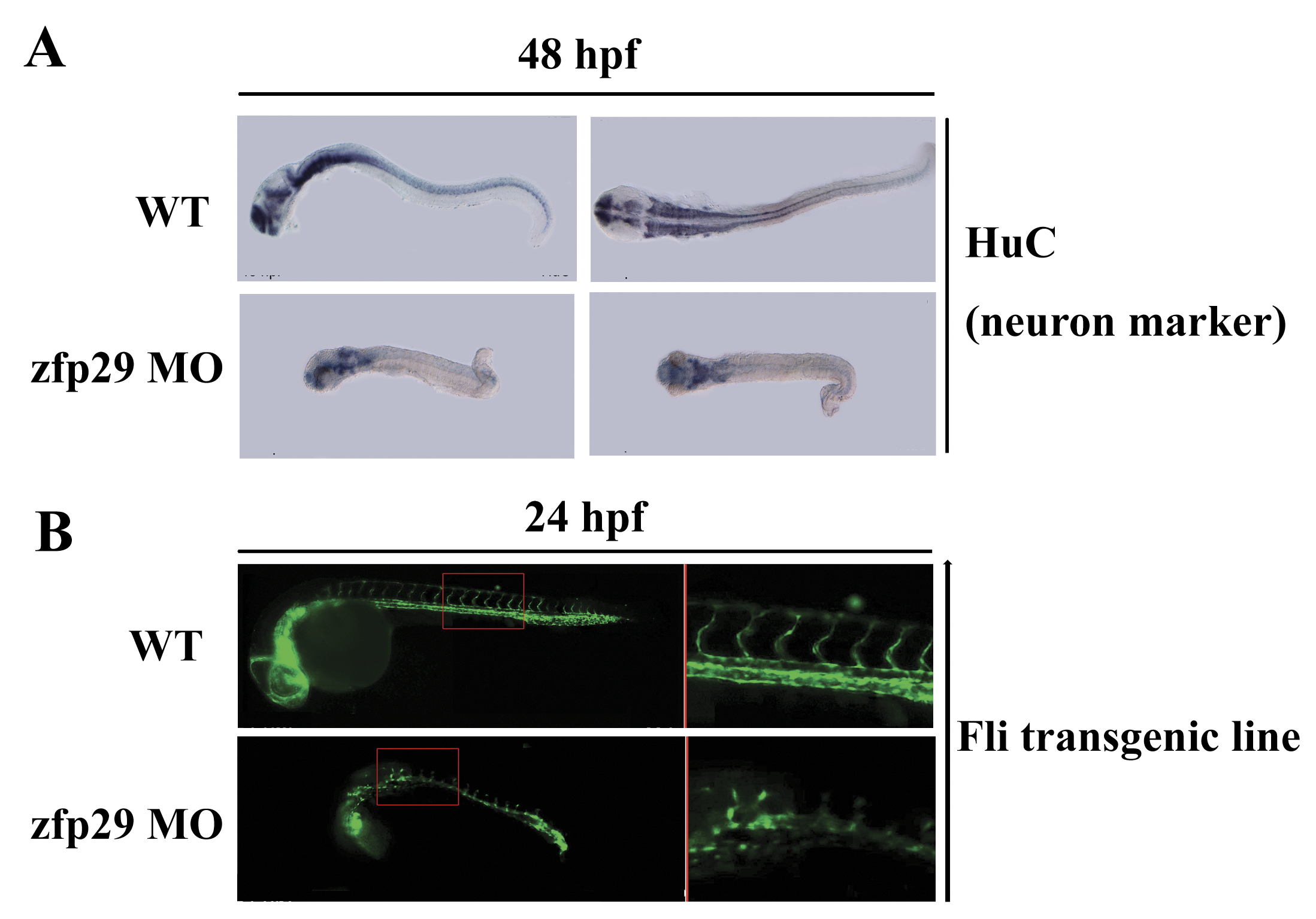

Supplement: Figure S6 — Expression of HuC and blood vessel formation in zfp29 knockdown morphants. (A) Zebrafish embryos at 2-cell stage were uninjected or injected with zfp29 MO and then probed with DIG-labeled HuC probe for in situ hybridization at 24 hpf. (B) Two-cell embryos of Fli transgenic line were injected with control and zfp29 MO and then fixed for detection of green fluorescence protein expression. (TIF) [file pone.0033538.s006.tif]
