# Supplementary material for: Disruption of Murine mp29/Syf2/Ntc31 Gene Results in Embryonic Lethality with Aberrant Checkpoint Response
Source: PLoS One. 2012 Mar 20;7(3):e33538. doi: 10.1371/journal.pone.0033538 (PMC3308990; doi:10.1371/journal.pone.0033538)
Supplement: Table S1 — List of mp29 associated proteins identified by Mass spectrometry analysis. (DOCX) [file pone.0033538.s007.docx]

| Pre-mRNA processing splicing factor 8 |
| --- |
| U5 small nuclear ribonucleoprotein 200 kDa helicase |
| 60S ribosomal protein L6 |
| Heterogeneous nuclear ribonucleoproteins A2/B1 |
| ATP dependent RNA helicase A |
| Vimentin |
| Major vault protein |
| Heterogeneous nuclear ribonucleoprotein U |
| Heterogeneous nuclear ribonucleoproteins A1 |
| 60S ribosomal protein L8 |
| Ankyrin repeat domain-containing protein 12 |
